# Supplementary material for: SARS-CoV-2 nsp3 and nsp4 are minimal constituents of a pore spanning replication organelle
Source: Nat Commun. 2023 Nov 30;14:7894. doi: 10.1038/s41467-023-43666-5 (PMC10689437; doi:10.1038/s41467-023-43666-5)
Supplement: Supplementary file 1 — Supplementary information [file 41467_2023_43666_MOESM1_ESM.pdf]

Supplementary Information

SARS-CoV-2 nsp3 and nsp4 are minimal constituents of a pore spanning replication organelle

Authors: Liv Zimmermann[1], Xiaohan Zhao[1], Jana Makroczyova[1], Moritz Wachsmuth-Melm[1], Vibhu Prasad[2], Zach Hensel[3], Ralf Bartenschlager[2, 4, 5], Petr Chlanda[1]*

*Corresponding author. Email: petr.chlanda@bioquant.uni-heidelberg.de

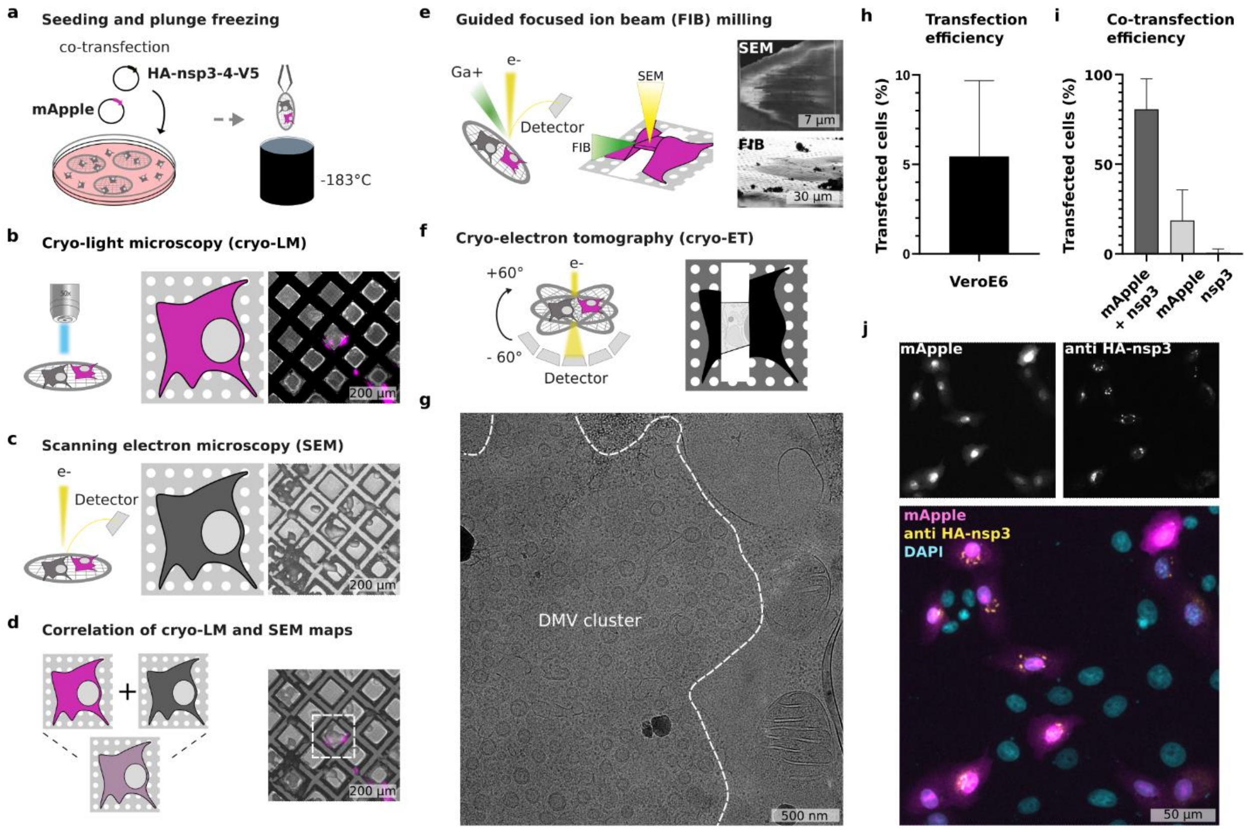

Supplementary Fig. 1.

***In situ* cryo-correlative light and electron microscopy workflow for guided cryo-focused FIB milling.** **a** VeroE6 cells were seeded on gold EM grids and transfected with plasmids encoding for SARS-CoV-2 nsp3-4 proteins and a fluorescent marker (mApple). Cells on grids were plunge frozen in liquid ethane. **b** The grid was mapped in a cryo-LM. **c** Inside a cryo-FIB/SEM microscope, a high resolution SEM image was acquired. **d** The cryo-LM and SEM maps were correlated to find transfected cells with the fluorescent mApple signal. **e** The mApple positive cells were FIB-milled. **f** The lamella was mapped by cryo-TEM (**g**) and tilt series were acquired on sites of interest. **b-f** Edited from [43]. **g** Map of a lamella containing a DMV cluster (borders of the cluster are indicated with dashed lines). **h** Transfection efficiency 24 hpt in VeroE6 cells (n = 3472 cells). **i** Co-transfection efficiency of the two constructs (n = 186 transfected cells). **h-i** Data are presented as mean with SD. Data from one experiment. **j** Co-transfection of plasmids encoding HA-nsp3-4-V5 and mApple (24 hpt). Nsp3 was detected by indirect immunofluorescence using an anti-HA antibody.

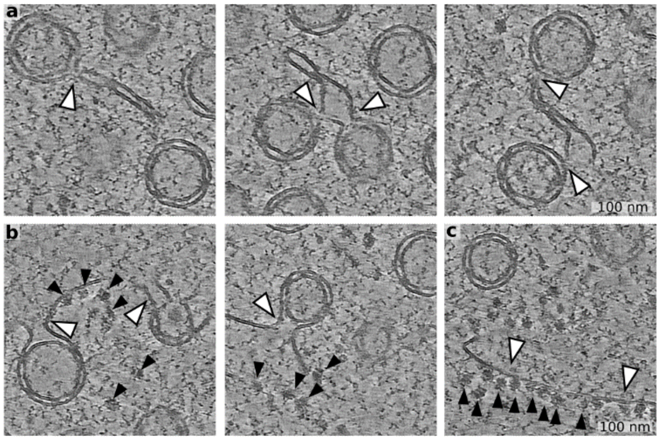

Supplementary Fig. 2.

Gallery of DMCs, DMV-ER connections and ER cisternae with ribosomes in close proximity to DMV network. a Examples of DMCs containing pores interconnecting several DMVs indicated by white arrowheads. **b** DMVs connected to ER indicated by white arrowheads. Ribosomes are indicated by black arrowheads. **c** ER cisternae indicated by white arrowheads and ribosomes indicated by black arrowheads directly next to DMVs.

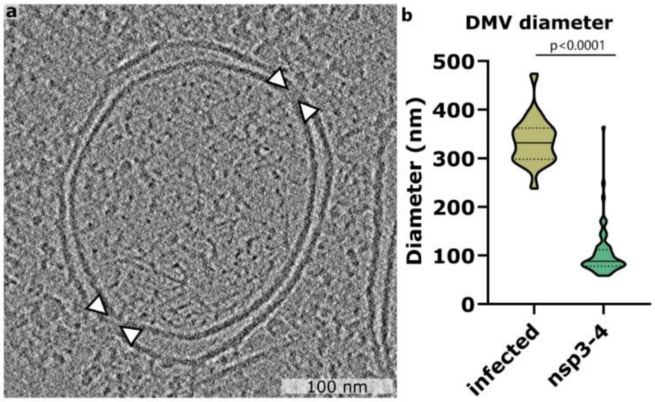

Supplementary Fig. 3.

Comparison of DMV diameter in infected and nsp3-4 transfected cells. a Slice of a tomogram showing a DMV found in SARS-CoV-2 infected and chemically fixed VeroE6 cells (dataset of tomograms from a previous publication[10]). Pores are highlighted by white arrowheads. **b** Plot showing the longest inner diameter of DMVs in SARS-CoV-2 infected (n = 20, 16 hpi) and in nsp3-4 transfected cells (n = 62, 16 hpt). Data is shown as a Violin plot indicating the median, 25% and 75% quartiles. Unpaired two-tailed t-test showed significant differences.

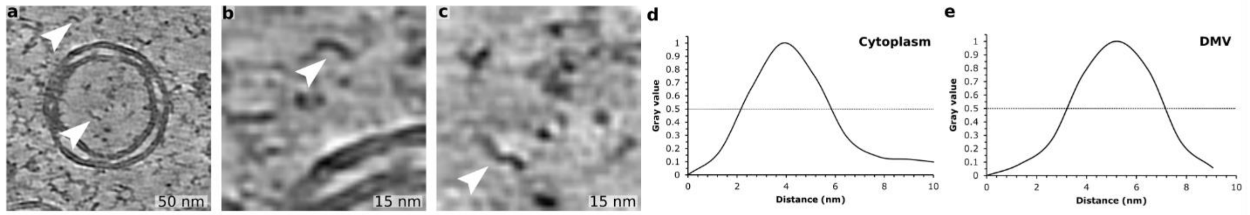

Supplementary Fig. 4.

Measurements of filaments found outside of DMVs and in the DMV lumen in VeroE6 cells.

a DMV found in nsp3-4 transfected cell. Indicated are filamentous structures outside and inside the DMV. **b** Magnified view of **a** shows a filamentous structure outside of DMV. **c** Magnified view of **a** shows a filamentous structure inside DMV. **d** Line density profile of filament outside of DMV was generated in ImageJ/FIJI and shows a diameter of 3.65 nm. **e** Line density profile of filament in the DMV lumen shows a diameter of 3.94 nm.

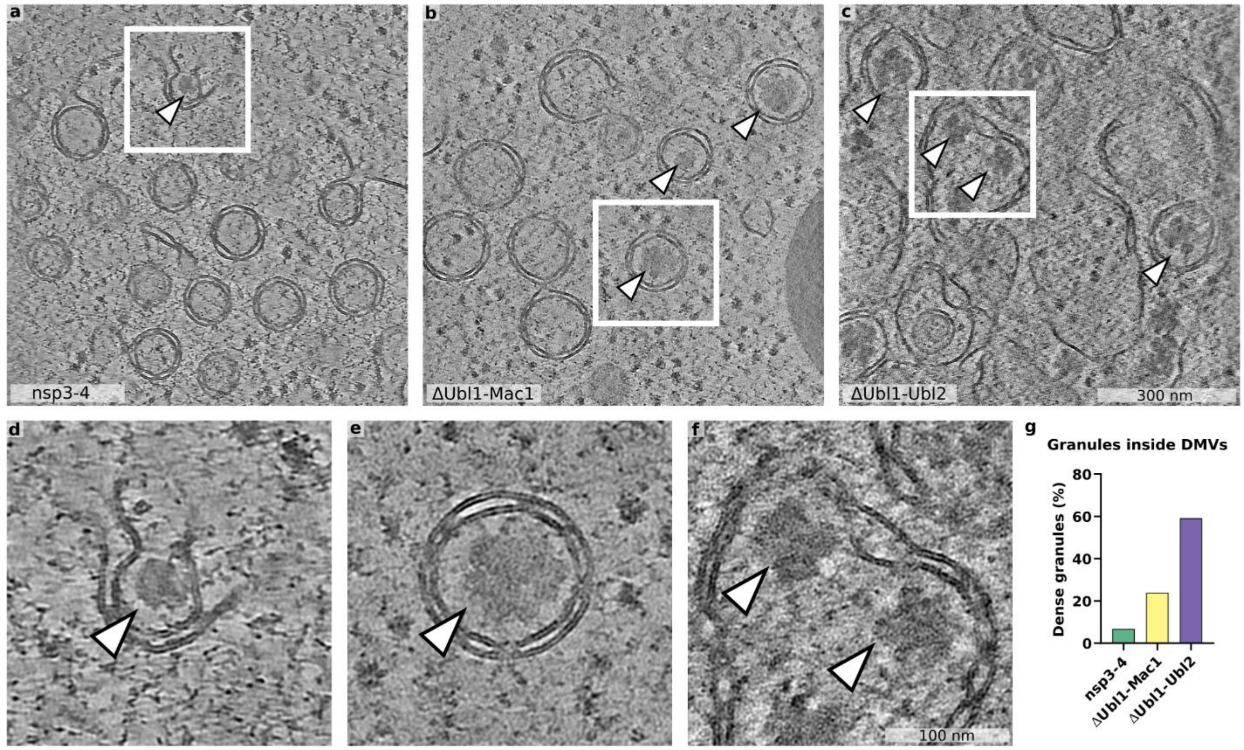

Supplementary Fig. 5.

Granular density of unknown origin localized inside DMVs. a-c Averaged slices of tomograms of VeroE6 cells transfected with nsp3-4 (**a**), ΔUbl1-Mac1 (**b**) and ΔUbl1-Ubl2 (**c**) and processed at 18-24 hpt. **d-f** Averaged slices of tomograms showing the magnified views highlighted by a white rectangle in (**a-c**). Granular densities of unknown origin are indicated by white arrowheads. **g** Distribution of DMVs filled with granular densities compared to the total amount of DMVs in nsp3-4, ΔUbl1-Mac1 and ΔUbl1-Ubl2 samples.

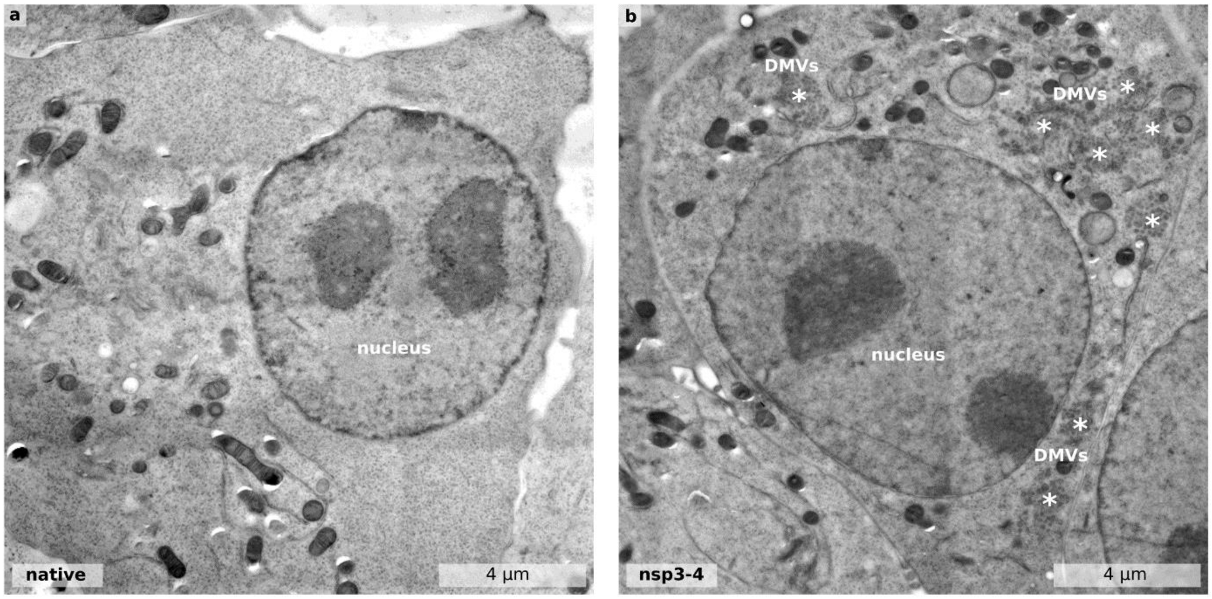

Supplementary Fig. 6.

DMV clusters distribute in cytoplasm. a Thin-section EM images of native HEK293T cells. **b** HEK293T cells transfected with HA-nsp3-4-V5. DMV clusters vary in size and measure up to several microns. DMV clusters are indicated with white asterisks.

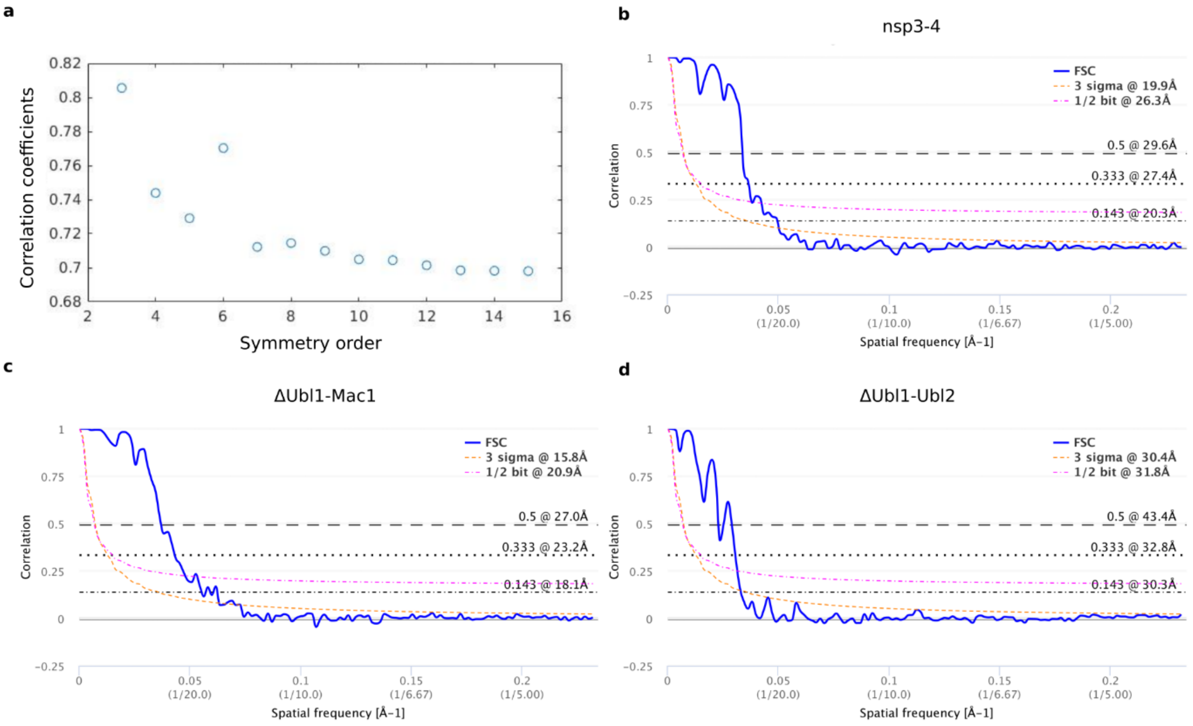

Supplementary Fig. 7.

Fourier shell correlation curves from subtomogram averages upon implying sixfold symmetry. a Correlation coefficients between unsymmetrized and symmetrized averages. **b** Fourier shell correlation curve from nsp3-4 pores showing a resolution of around 20.3Å at 0.143 criterion. **c** Fourier shell correlation curve from ΔUbl1-Mac1 pores showing a resolution of around 18.1Å at 0.143 criterion. **d** Fourier shell correlation curve from ΔUbl1-Ubl2 pores showing a resolution of around 30.3Å at 0.143 criterion.

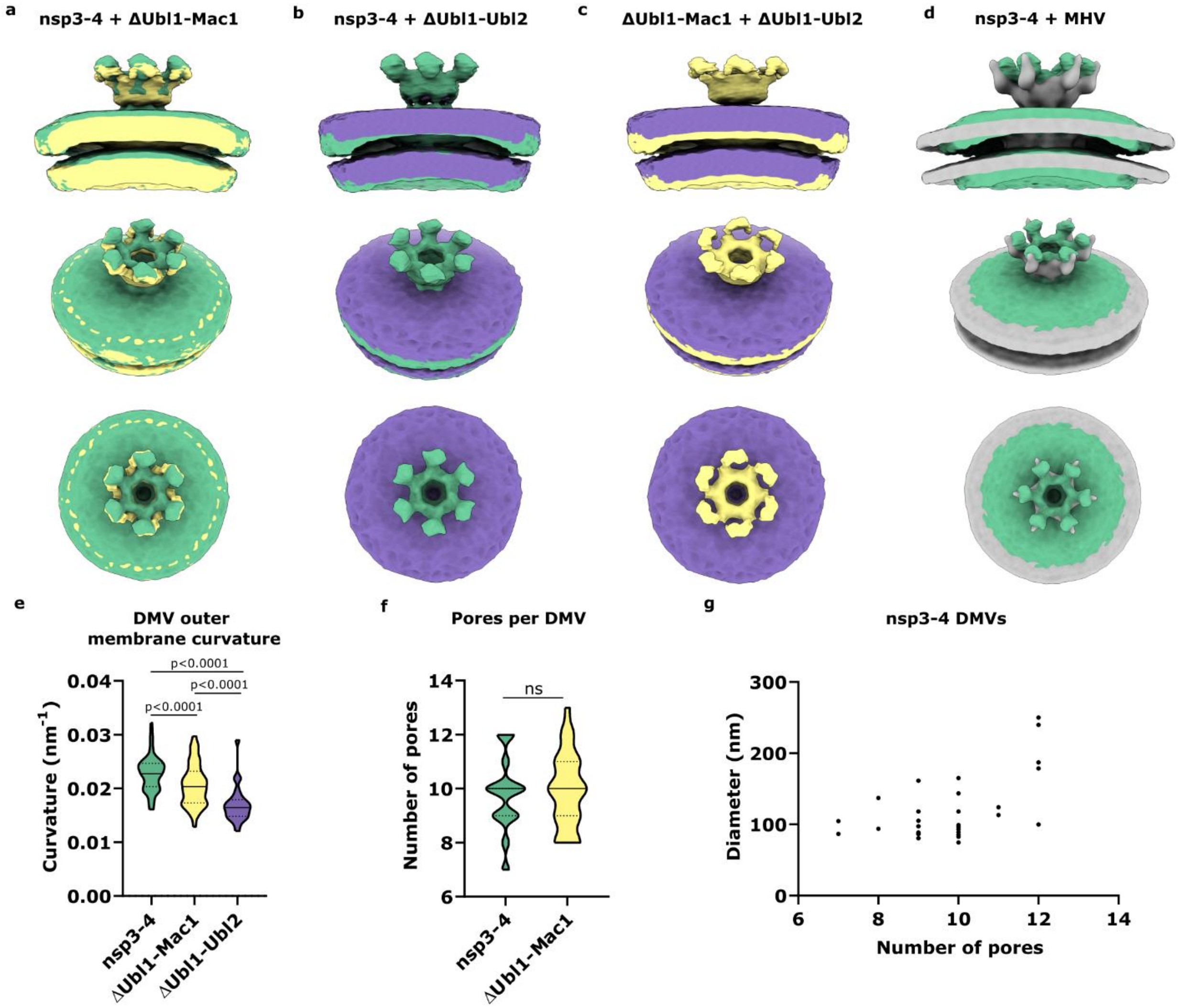

Supplementary Fig. 8.

Comparison of isosurfaces and additional measurements characterizing the DMVs. a Isosurface of nsp3-4 pore compared to the isosurface of ΔUbl1-Mac1 pore showing partially collapsed prongs in ΔUbl1-Mac1 pore and differences in membrane curvature. **b** Isosurface of nsp3-4 pore compared to the isosurface of ΔUbl1-Ubl2 pore showing the loss of the prongs in ΔUbl1-Ubl2 pore and differences in membrane curvature. **c** Isosurface of ΔUbl1-Mac1 pore compared to the isosurface of ΔUbl1-Ubl2 pore showing the

loss of the prongs in ΔUbl1-Ubl2 pore and differences in membrane curvature. **d** Isosurface of nsp3-4 pore (surface level 0.375) compared to the isosurface of DMV pore of MHV infected cells (surface level 2.19) showing differences in prongs of pores and differences in membrane curvature. **e** The outer membrane curvature of DMV was determined using the osculating circle method (nsp3-4: n = 103; ΔUbl1-Mac1: n = 103; ΔUbl1-Ubl2: n = 26) **f** Number of pores per DMV in nsp3-4 (n = 31), ΔUbl1-Mac1 (n = 31). Data is shown as a Violin plot indicating the median, 25% and 75% quartiles. Unpaired two-tailed t-test showed significant differences. **g** Inner DMV diameter and number of pores per DMV do not correlate (R^2=0.2915).

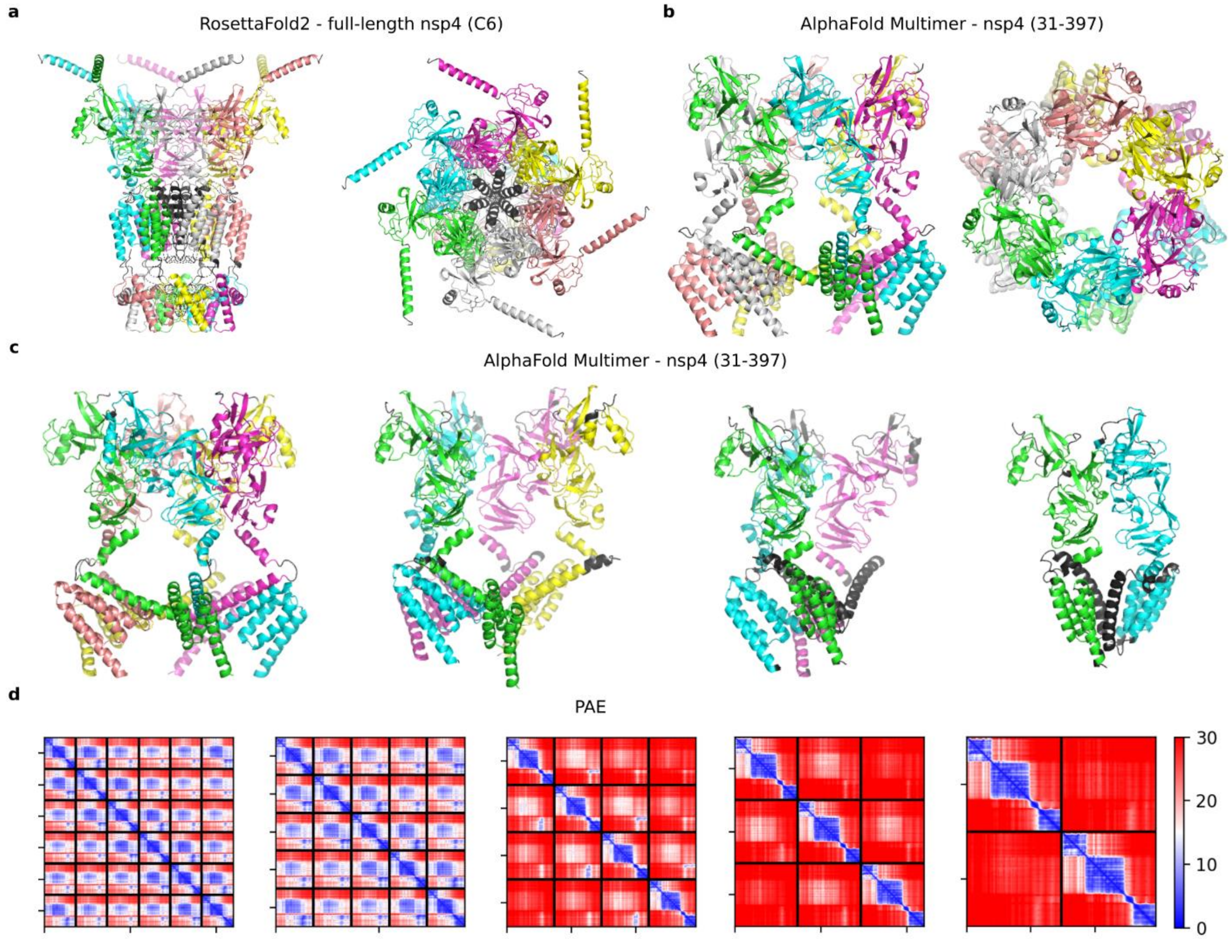

Supplementary Fig. 9.

Prediction of nsp4 with RosettaFold2 and AlphaFold Multimer. a RosettaFold2 prediction of full-length nsp4 enforcing C6 symmetry. Residues 268-315 linking nsp4 luminal and TM domains lacks a high confidence prediction, as do residues 393-414 linking TM and C-terminal domains. **b** Unconstrained interference of six copies of nsp4 residues 31-397 with AlphaFold Multimer predicts a hexamer. **a-b** Left: side view, right: top view. **c** Structures of best scoring complexes for pentamer, tetramer, trimer and dimer predicted with AlphaFold Multimer. **a-c** Residues are colored by chain except for those with pLDDT < 50

colored in dark gray. **d** Pair alignment error (PAE) of predictions for nsp4 multimers (31-397) shows that interaction between subunits is predicted with higher confidence for hexameric and pentameric complexes. Left-right: PAE for hexamer, pentamer, tetramer, trimer and dimer. Scale bar in Å.

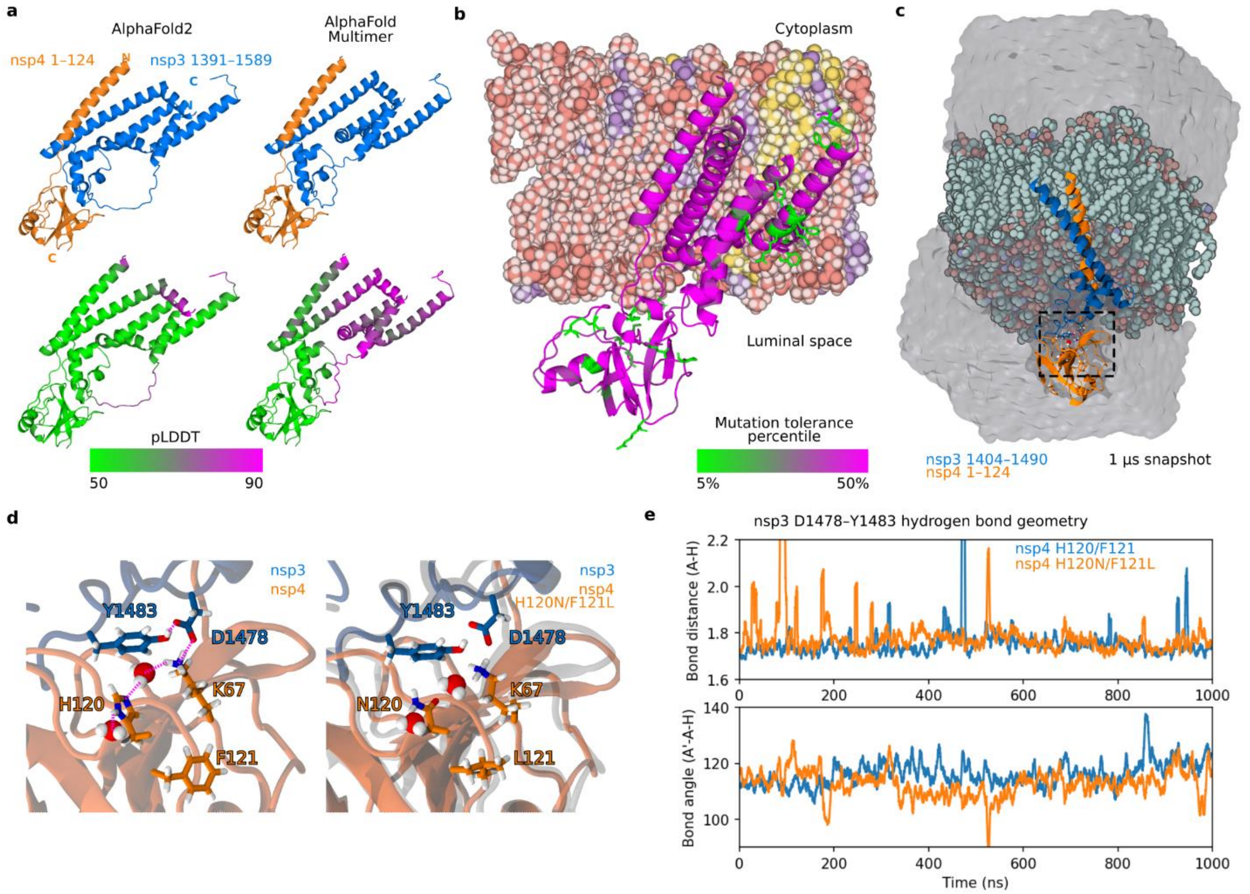

Supplementary Fig. 10.

Prediction, conservation, and dynamics of nsp3-nsp4 complex at the DMV outer membrane. a Structure predictions by AlphaFold2 and AlphaFold-Multimer and their pLDDT values are shown for all predicted residues nsp3 1391–1589 and nsp4 1–124. **b** The average of the logarithm of the tolerance to mutation for the first two nucleotides in each codon is mapped to the AlphaFold2 structure prediction, scaled from the 5th to 50th percentile of values for all of ORF1a. Residues in the bottom 10th percentile for mutation tolerance are shown as sticks and include an nsp4 disulfide bond, buried hydrophobic residues, the hydrogen bond network at the nsp3-nsp4 interface, and a cluster of nsp3 residues that may contribute to membrane curvature and/or pore oligomerization. Alignment to the lipid bilayer is based on coordinates of nsp4 36–124 after 1 µs molecular dynamics. POPC, DOPE, and SAPI lipids are colored pink, purple and yellow, respectively. Cytoplasmic and luminal faces of the bilayer are based on predicted membrane topology. **c** Tilted view of all-atom molecular dynamics system for simulated chains nsp3 1404-1490 and

nsp4 1-124; although nsp3 and nsp4 are rendered separately, helices are buried in the bilayer. Non-hydrogen bilayer atoms are shown and colored by atom name. **d** The region highlighted in **c** is shown for the 1-μs timepoint of nsp4 wild-type and H120N/F121L simulations. Structures are aligned to the Cα positions of labeled residues and labels are positioned identically to facilitate comparison. H120N disrupts the hydrogen bond network at the nsp3-nsp4 interface formed by nsp3 D1478, nsp3 Y1483, and nsp4 K67, with nsp3 Y1483 displaced and less optimal geometry for hydrogen bonds to a water molecule linking nsp4 K67 and nsp4 H120. The wild-type conformation is reproduced in gray on the right to illustrate deformation by the buried, hydrophobic F121L mutation. **e** 5-ns moving averages of hydrogen bonding geometry between nsp3 D1478 and nsp3 Y1483 show instability in the H120N/F121L simulation for the first ~300 ns followed by a decrease of 4.2 ° in the median bond angle in the final 500 ns of simulation.

Step	T start	T end	Slope	Time	Reagent	Transfer	Agitation	UV
1	-90°C	-90°C		48 h	0.1% UA	stay	off	off
2	-90°C	-45°C	5 °C/h	9 h	0.1% UA	stay	off	off
3	-45°C	-45°C		5 h	0.1% UA	stay	off	off
4	-45°C	-45°C		10 min	Acetone	exch/fill	off	off
5	-45°C	-45°C		10 min	Acetone	exch/fill	off	off
6	-45°C	-45°C		10 min	Acetone	exch/fill	off	off
7	-45°C	-45°C		4 h	10% Lowicryl	mix	on	off
8	-45°C	-45°C		4 h	25% Lowicryl	mix	on	off
9	-45°C	-35°C	2.5 °C/h	4 h	50% Lowicryl	mix	on	off
10	-35°C	-35°C	2.5 °C/h	4 h	75% Lowicryl	mix	on	off
11	-35°C	-35°C		10 h	100% Lowicryl	exch/fill	off	off
12	-35°C	-35°C		10 h	100% Lowicryl	exch/fill	off	off
13	-35°C	-35°C		10 h	100% Lowicryl	exch/fill	off	off
14	-35°C	-35°C		48 h	100% Lowicryl	stay	off	on
15	-35°C	20°C	5	9 h	100% Lowicryl	stay	off	on
16	20°C	20°C		48 h	100% Lowicryl	stay	off	on
17	20°C	20°C		72 h	100% Lowicryl	stay	off	off

Supplementary Table 1.
Automated freeze substitution program. UA: uranyl acetate.

Parameters	Round 1	Round 2	Round 3	Round 4	Round 5	Round 6
Iterations	1	1	1	1	1	1
References	1	1	1	1	1	1
Cone aperture [°]	360	180	90	45	24	12
Cone Sampling [°]	120	60	30	15	8	4
Azymuth rotation range [°]	360	180	90	45	24	12
Azymuth rotation sampling [°]	120	60	30	15	8	4
Refine	5	5	5	5	5	5
Refine factor	2	2	2	2	2	2
High pass filter [pixel]	2	2	2	2	2	2
Low pass filter [pixel]	32	32	64	64	80	128
Symmetry	C1/C6	C1 / C6	C1 / C6	C1 / C6	C1 / C6	C1 / C6
Particle dimensions	128	128	128	128	128	256
Shift limits in X, Y and Z [pixel]	30, 30, 30	20, 20, 20	12, 12, 12	8, 8, 8	4, 4, 4	2, 2, 2
Shift limiting way	2	2	2	2	2	2
Separation in tomogram	1	1	1	1	1	1

Supplementary Table 2.
Numerical parameters for subtomogram averaging calculation in Dynamo.

Construct	Number of lamellae	Number of tomograms	Number of particles	Resolution (0.143 Criterion)	Resolution (0.5 Criterion)
**Nsp3-4**	5	8	325	20.3 Å	29.6 Å
**Nsp3-4 ΔUB1-MAC1**	4	7	1099	18.1 Å	27.0 Å
**Nsp3-4 ΔUB1-UBl2**	3	8	94	30.3 Å	43.4 Å

Supplementary Table 3.
Number of particles used for subtomogram averaging and attained resolution.
