# Supplementary material for: SARS-CoV-2 nsp3 and nsp4 are minimal constituents of a pore spanning replication organelle
Source: Nat Commun. 2023 Nov 30;14:7894. doi: 10.1038/s41467-023-43666-5 (PMC10689437; doi:10.1038/s41467-023-43666-5)
Supplement: Supplementary file 3 — Description of Additional Supplementary Files [file 41467_2023_43666_MOESM3_ESM.docx]

**Description of Additional Supplementary Files**

**Supplementary Movie 1:** Tomogram of DMVs formed in VeroE6 cells expressing nsp3-4 construct. DMV cluster consists of DMVs which are connected to each other and are derived from ER membranes which are decorated with ribosomes (also see Supplementary Fig. 2). DMVs are spherical and contain pores. Scale bar: 100 nm.

**Supplementary Movie 2:** Tomogram of DMVs formed in VeroE6 cells expressing ΔUbl1- Mac1 construct. DMVs are spherical and contain pores. Scale bar: 100 nm.

**Supplementary Movie 3:** Tomogram of DMVs formed in VeroE6 cells expressing ΔUbl1- Ubl2 construct. DMV cluster consists of only partially closed DMVs and completely closed DMVs with ovoidal shape. DMVs contain pores but the crown of the pores is not visible anymore. Scale bar: 100 nm.

**Supplementary Movie 4:** Tomogram of DMVs formed in VeroE6 cells expressing GG>AA construct. Membranes are paired to form DMV-like structure with much larger diameter but membranes lack pores. Scale bar: 200 nm.

**Supplementary Data 1:** Protein sequences of nsp3-4 constructs.
