# Supplementary material for: SARS-CoV-2 nsp3 and nsp4 are minimal constituents of a pore spanning replication organelle
Source: Nat Commun. 2023 Nov 30;14:7894. doi: 10.1038/s41467-023-43666-5 (PMC10689437; doi:10.1038/s41467-023-43666-5)
Supplement: Supplementary file 9 — Reporting Summary [file 41467_2023_43666_MOESM9_ESM.pdf]

nature portfolio

Corresponding author(s): Petr Chlanda
Last updated by author(s): Nov 4, 2023

Reporting Summary

Nature Portfolio wishes to improve the reproducibility of the work that we publish. This form provides structure for consistency and transparency in reporting. For further information on Nature Portfolio policies, see our Editorial Policies and the Editorial Policy Checklist.

Statistics

For all statistical analyses, confirm that the following items are present in the figure legend, table legend, main text, or Methods section.

n/a	Confirmed	
☐	☒	The exact sample size (*n*) for each experimental group/condition, given as a discrete number and unit of measurement
☐	☒	A statement on whether measurements were taken from distinct samples or whether the same sample was measured repeatedly
☐	☒	The statistical test(s) used AND whether they are one- or two-sided<br>*Only common tests should be described solely by name; describe more complex techniques in the Methods section.*
☒	☐	A description of all covariates tested
☒	☐	A description of any assumptions or corrections, such as tests of normality and adjustment for multiple comparisons
☐	☒	A full description of the statistical parameters including central tendency (e.g. means) or other basic estimates (e.g. regression coefficient) AND variation (e.g. standard deviation) or associated estimates of uncertainty (e.g. confidence intervals)
☐	☒	For null hypothesis testing, the test statistic (e.g. *F*, *t*, *r*) with confidence intervals, effect sizes, degrees of freedom and *P* value noted<br>*Give P values as exact values whenever suitable.*
☒	☐	For Bayesian analysis, information on the choice of priors and Markov chain Monte Carlo settings
☒	☐	For hierarchical and complex designs, identification of the appropriate level for tests and full reporting of outcomes
☒	☐	Estimates of effect sizes (e.g. Cohen's *d*, Pearson's *r*), indicating how they were calculated

Our web collection on statistics for biologists contains articles on many of the points above.

Software and code

Policy information about availability of computer code

Data collection	Cryo-EM data collection: SerialEM 4.0, MAPS Software, Autolamella script available at https://github.com/DeMarcoLab/autolamella; Fluorescence microscopy data collection: Leica TCS SP8 acquisition software; LAS X Navigator software; Western Blot: Azure 400 Imaging System software
Data analysis	FIJI; IMOD; Dynamo; CLEM/Stitch TileScan plugin available at https://github.com/Chlanda-Lab/cryoCLEM; Nearest neighbor script (10.5281/zenodo.10066867), Structure prediction and MD simulation: DeepTMHMM, AplhaFold2, ColabFold, AlphaFold-Multimer, CHARMM-GUI Membrane Builder

For manuscripts utilizing custom algorithms or software that are central to the research but not yet described in published literature, software must be made available to editors and reviewers. We strongly encourage code deposition in a community repository (e.g. GitHub). See the Nature Portfolio guidelines for submitting code & software for further information.

Data

Policy information about availability of data

All manuscripts must include a data availability statement. This statement should provide the following information, where applicable:

- Accession codes, unique identifiers, or web links for publicly available datasets
- A description of any restrictions on data availability
- For clinical datasets or third party data, please ensure that the statement adheres to our policy

Electron tomography data have been deposited to the Electron Microscopy Data Bank under accession codes EMD-15925 (tomogram of nsp3-4 induced DMVs in Fig.1), EMD-15926 (tomogram of ΔUbl1-Ubl2 induced pores in Fig.3), EMD-15927 (tomogram of ΔUbl1-Mac1 induced pores in Fig.3), EMD-15928 (tomogram of GG>AA induced pores in Fig.3), EMD-15929 (tomogram of nsp3-4 induced pores in Fig.3), EMD-15963 (subtomogram average of nsp3-4 pore in Fig.1), EMD-15964 (subtomogram average of ΔUbl1-Mac1 pore in Fig.4), EMD-15965 (subtomogram average of ΔUbl1-Ubl2 pore in Fig.4). Files for the MD trajectories will be available at Zenodo (DOI: 10.5281/zenodo.10069883). Source data are provided with this paper.

Research involving human participants, their data, or biological material

Policy information about studies with human participants or human data. See also policy information about sex, gender (identity/presentation), and sexual orientation and race, ethnicity and racism.

Reporting on sex and gender	No human research participants were involved in this study.
Reporting on race, ethnicity, or other socially relevant groupings	*Please specify the socially constructed or socially relevant categorization variable(s) used in your manuscript and explain why they were used. Please note that such variables should not be used as proxies for other socially constructed/relevant variables (for example, race or ethnicity should not be used as a proxy for socioeconomic status).*<br>*Provide clear definitions of the relevant terms used, how they were provided (by the participants/respondents, the researchers, or third parties), and the method(s) used to classify people into the different categories (e.g. self-report, census or administrative data, social media data, etc.)*<br>*Please provide details about how you controlled for confounding variables in your analyses.*
Population characteristics	*Describe the covariate-relevant population characteristics of the human research participants (e.g. age, genotypic information, past and current diagnosis and treatment categories). If you filled out the behavioural & social sciences study design questions and have nothing to add here, write "See above."*
Recruitment	*Describe how participants were recruited. Outline any potential self-selection bias or other biases that may be present and how these are likely to impact results.*
Ethics oversight	*Identify the organization(s) that approved the study protocol.*

Note that full information on the approval of the study protocol must also be provided in the manuscript.

Field-specific reporting

Please select the one below that is the best fit for your research. If you are not sure, read the appropriate sections before making your selection.

☒ Life sciences ☐ Behavioural & social sciences ☐ Ecological, evolutionary & environmental sciences

For a reference copy of the document with all sections, see nature.com/documents/nr-reporting-summary-flat.pdf

Life sciences study design

All studies must disclose on these points even when the disclosure is negative.

Sample size	Confocal microscopy: 10-14 cells were imaged and analyzed for collocalization. Since no large variation within the population was observed we concluded that the sample size is representative and sufficient.<br>Thin section EM sample size was limited by transfection efficiency. Representative images were selected from at least 5 cells showing the phenotype.<br>Cryo-ET tomography data and number of particles is provided in the Table S3. In this study 3-5 cells and 7-8 high quality tomograms per sample were used for subtomogram averaging which yielded 94-1099 particles. Here more particles could be used to further increased the resolution, however, the current number of particles was sufficient to obtain comparable maps.
Data exclusions	No data were excluded in this study except of cryo-ET data which was obtained from lamella which were too thick and not suitable for subtomogram averaging.
Replication	Western blots are representative data from three biologically independent experiments. Confocal microscopy data is from one experiment. Thin-section EM data is from one experiment. Cryo-ET data of nsp3-4 construct is representative data from two biologically independent experiments. Other cryo-ET data is from one experiment.
Randomization	Randomization was not relevant in this study as the phenotypes in EM experiments were very obvious during data analysis.

Blinding	Blinding was not applied in this study as no formal hypothesis was proposed and tested.

Reporting for specific materials, systems and methods

We require information from authors about some types of materials, experimental systems and methods used in many studies. Here, indicate whether each material, system or method listed is relevant to your study. If you are not sure if a list item applies to your research, read the appropriate section before selecting a response.

Materials & experimental systems

n/a	Involved in the study
☐	☒ Antibodies
☐	☒ Eukaryotic cell lines
☒	☐ Palaeontology and archaeology
☒	☐ Animals and other organisms
☒	☐ Clinical data
☒	☐ Dual use research of concern
☒	☐ Plants

Methods

n/a	Involved in the study
☒	☐ ChIP-seq
☒	☐ Flow cytometry
☒	☐ MRI-based neuroimaging

Antibodies

Antibodies used	To detect the HA-tag and V5-tag by confocal microscopy and Western blot analysis, a rabbit anti-HA (Invitrogen, 71-5500), mouse anti-V5 (Santa Cruz Biotechnology, sc-271944) and mouse anti-GAPDH antibodies (Santa Cruz Biotechnology, sc-47724) were used as primary antibodies, respectively. The secondary antibodies Alexa Fluor 546 goat anti-rabbit (Invitrogen, A11010) and Alexa Fluor 488 goat anti-mouse (Invitrogen, A11029) were used for confocal microscopy. The secondary antibodies mouse anti-rabbit IgG-HRP (Santa Cruz Biotechnology, sc-2357) and anti-mouse IgG BP-HRP (Santa Cruz Biotechnology, sc-516102) were used for Western blot analysis. Dilutions for microscopy: primary antibody 1:200, secondary antibody 1:500. Western blot: anti-HA 1:200, anti-V5 and anti-GAPDH 1:1000, secondary antibody 1:1000.
Validation	Anti-HA tag, Invitrogen, 71-5500 antibody was validated by ThermoFisher Scientific company by immunofluorescence microscopy and by Western Blot analysis. Anti-V5 antibody (Santa Cruz Biotechnology, sc-271944) was validated by Western Blot analysis by SCB company. Anti-GAPDH (Santa Cruz Biotechnology, sc-47724) was validated by Western Blot analysis and by immunofluorescence microscopy in the SCB company.

Eukaryotic cell lines

Policy information about cell lines and Sex and Gender in Research

Cell line source(s)	HEK293T (293T ECACC, 1202201) cells were purchased from Sigma-Aldrich. VeroE6 cells were purchased from American Type Culture Collection (ATCC; Catalogue #CRL-1586).
Authentication	The cell line were obtained from companies which guarantee cell line authenticity.
Mycoplasma contamination	Mycoplasma test is performed every 3 months and cells used in this study were never positive for mycoplasma.
Commonly misidentified lines (See ICLAC register)	Vero and HEK cells are among commonly misidentified cells. However, in this study authentic HEK-293T and VeroE6 cells were purchased from Sigma Aldrich and ATCC, respectively.

Plants

Seed stocks	*Report on the source of all seed stocks or other plant material used. If applicable, state the seed stock centre and catalogue number. If plant specimens were collected from the field, describe the collection location, date and sampling procedures.*
Novel plant genotypes	*Describe the methods by which all novel plant genotypes were produced. This includes those generated by transgenic approaches, gene editing, chemical/radiation-based mutagenesis and hybridization. For transgenic lines, describe the transformation method, the number of independent lines analyzed and the generation upon which experiments were performed. For gene-edited lines, describe the editor used, the endogenous sequence targeted for editing, the targeting guide RNA sequence (if applicable) and how the editor was applied.*
Authentication	*Describe any authentication procedures for each seed stock used or novel genotype generated. Describe any experiments used to assess the effect of a mutation and, where applicable, how potential secondary effects (e.g. second site T-DNA insertions, mosiacism, off-target gene editing) were examined.*
